# Apoptosis-promoting properties of miR-3074-5p in MC3T3-E1 cells under iron overload conditions

**DOI:** 10.1186/s11658-021-00281-w

**Published:** 2021-08-16

**Authors:** Yi Feng, Pei-Yan He, Wei-Dong Kong, Wan-Jing Cen, Peng-Lin Wang, Chang Liu, Wu Zhang, Shu-Shu Li, Jian-Wei Jiang

**Affiliations:** 1 Department of Orthodontics, The First Affiliated Hospital of Jinan University, No.613 Huangpu Road West, Guangzhou, 510630 China; 2 Department of Biochemistry, Basic Medical College, Jinan University, No.601 Huangpu Road West, Guangzhou, 510632 China; 3 Department of Orthodontics, School of Stomatology, Jinan University, Guangzhou, China

**Keywords:** Iron overload, Osteoporosis, Apoptosis, MC3T3-E1 cells, miRNA-3074-5p

## Abstract

**Background:**

Iron overload can promote the development of osteoporosis by inducing apoptosis in osteoblasts. However, the mechanism by which miRNAs regulate apoptosis in osteoblasts under iron overload has not been elucidated.

**Method:**

The miRNA expression profile in MC3T3-E1 cells under iron overload was detected by next generation sequencing. qRT-PCR was used to determine the expression of miR-3074-5p in MC3T3-E1 cells under iron overload. The proliferation of MC3T3-E1 cells was tested using CCK-8 assays, and apoptosis was measured using flow cytometry. The miRanda and TargetScan databases were used to predict the target genes of miR-3074-5p. Interaction between miR-3074-5p and the potential target gene was validated by qRT-PCR, luciferase reporter assay and western blotting.

**Results:**

We found that iron overload decreased the cell viability and induced apoptosis of MC3T3-E1 cells. The results of next generation sequencing analysis showed that miR-3074-5p expression was significantly increased in MC3T3-E1 cells under iron overload conditions, which was confirmed by further experiments. The inhibition of miR-3074-5p attenuated the apoptosis of iron-overloaded MC3T3-E1 cells. Furthermore, the expression of Smad4 was decreased and was inversely correlated with miR-3074-5p expression, and overexpression of Smad4 partially reversed the viability inhibition of iron-overloaded MC3T3-E1 cells by relieving the suppression of ERK, AKT, and Stat3 phosphorylation, suggesting its regulatory role in the viability inhibition of iron-overloaded MC3T3-E1 cells. The luciferase reporter assay results showed that Smad4 was the target gene of miR-3074-5p.

**Conclusion:**

miR-3074-5p functions as an apoptosis promoter in iron-overloaded MC3T3-E1 cells by directly targeting Smad4.

**Supplementary Information:**

The online version contains supplementary material available at 10.1186/s11658-021-00281-w.

## Background

Iron is one of the essential nutrients for mammals. However, iron accumulation in tissues, which is characteristic of the elderly, is a risk factor for infection, osteoporosis, and several chronic diseases [1]. Osteoporosis is a systemic disease that causes reduced bone strength and an increased risk of fracture, which is caused by reduced bone mass and bone microstructure destruction. Fracturing is a serious consequence of osteoporosis, which can significantly increase the morbidity and mortality of patients, and cause considerable family and socioeconomic burdens [2]. Evidence from several studies has demonstrated that iron overload directly inhibits bone remodeling [3–5]. In a study of fifty patients with osteoporosis, 88% of them were iron overloaded, suggesting that iron overload may play a role in promoting the development and progression of osteoporosis [6]. The steady state of the skeletal system depends on the dynamic equilibrium between osteoblast-mediated osteogenesis and osteoclast-mediated bone destruction in bone metabolism. When the number or function of osteoblasts decreased, bone formation was less than resorption, resulting in loss of alveolar bone mass. Many in vivo and in vitro studies have suggested that iron overload could reduce bone formation and increase bone resorption, which is associated with pathological bone loss [7–9]. Cell studies in vitro have found that iron overload induced osteoblast apoptosis by increasing intracellular reactive oxygen species and activating the mitochondrial apoptosis pathway [10–12].

miRNAs are a class of endogenous non-coding single-stranded small RNAs with a length of approximately 20–22 nucleotides. These RNAs play a key role in cell metabolism, proliferation, autophagy, apoptosis, differentiation and other biological processes, and their expression changes are related to many human diseases [13–15]. Studies have shown that miRNAs participate in regulating apoptosis. Several apoptosis-related genes are regulated by miRNAs [16–18]. miR-1 promoted nitric oxide-induced apoptosis of MC3T3-E1 cells by targeting HSP-70 [19]. miR-214 alleviated apoptosis of H_2_O_2_-treated MC3T3-E1 cells by suppressing oxidative stress [20]. However, the mechanism by which miRNAs regulate apoptosis in MC3T3-E1 cells under iron overload has not been reported.

The present study used an iron overload model of MC3T3-E1 cells induced by a high concentration of ferric ammonium citrate (FAC) to simulate an environment of iron overload and analyzed the miRNA expression profiles in MC3T3-E1 cells under iron overload conditions. The results showed that increased miR-3074-5p due to iron overload caused apoptosis of MC3T3-E1 cells. Overexpression of miR-3074-5p inhibited Smad4 in MC3T3-E1 cells. Restoration of Smad4 rescued the effects of miR-3074-5p in FAC-treated MC3T3-E1 cells. The present data show the apoptosis-promoting properties of miR-3074-5p by regulating its downstream gene target Smad4 in MC3T3-E1 cells under iron overload in vitro.

## Methods

### Cell culture, drug treatment, and transfection

The murine preosteoblast cell line MC3T3-E1 was obtained from American Type Culture Collection (Manassas, VA, USA) and stored at the Department of Biochemistry, Medical College of Jinan University (Guangzhou, China). MC3T3-E1 cells were cultured in α-MEM (Gibco-BRL, Gaithersburg, MD, USA) containing 10% fetal bovine serum at 37 °C in an incubator with 5% carbon dioxide [21]. To simulate an in vitro environment of iron overload (iron overload conditions), cells were treated with 0, 0.6, 1.2, 1.8, 2.4 and 3.0 mM ferric ammonium citrate (FAC; Sangon, Shanghai, China). After exposure to FAC for specified times, all samples were collected for the following experiments. To overexpress and block miR-3074-5p in MC3T3-E1 cells, cells were transfected with miR-3074-5p mimic, inhibitor, and the negative control (Ribo, Guangzhou, China) separately at a working concentration of 100 nM using the Ribo FECT CP Transfection Kit (Ribo, Guangzhou, China) following the manufacturer’s guidelines. miRNA inhibitor is a chemically synthesized 21–23 nt oligonucleotide modified by 2ʹ-methoxy, which can specifically bind to mature miRNA to reduce its regulatory effect in cells. The sequences of mimic, mimic NC, inhibitor and inhibitor NC used are shown in Table 1. To overexpress Smad4 in MC3T3-E1 cells, the cells were transfected with pcDNA3.1-Smad4 and a pcDNA3.1-negative control using Lipofectamine 2000 (Invitrogen, Carlsbad, CA, USA). Intracellular expression of Smad4 was confirmed by western blot analysis.

**Table 1:** Sequences of mimic, mimic NC, inhibitor, and inhibitor NC

mimic/inhibitor/NC	Sequences
miR-3074-5p mimic	5′-GUUCCUGCUGAACUGAGCCAGUUGGCUCAGUUCAGCAGGAACUU-3′
mimic NC	Sense: 5′-UUCUCCGAACGUGUCACGUTT-3′ Antisense: 5′-ACGUGACACGUUCGGAGAATT-3′
miR-3074-5p inhibitor	5′-ACUGGCUCAGUUCAGCAGGAAC-3′
Inhibitor NC	5′-CAGUACUUUUGUGUAGUACAA-3′

### Cell viability measurement

Cell viability was detected using a CCK-8 assay (Sigma-Aldrich, St Louis, USA). MC3T3-E1 cells were cultured in 96-well plates at 3 × 10^3^ cells per well. After 24, 48 and 72 h of FAC treatment, the cell inhibition rate was determined with a CCK-8 assay following the manufacturer’s guidelines [22]. Cell inhibition rate = (1 − mean of OD values of the treatment group/mean of OD values of the control group) × 100%.

### Hoechst staining to detect apoptosis-related morphologic changes

MC3T3-E1 cells were cultured in 6-well plates at 5 × 10^4^ cells per well. After 24 h of 0 and 1.8 mM FAC treatment for 24 h, the cells were fixed. A Hoechst 33,258 staining kit (Beyotime, Shanghai, China) was used for cell staining [23]. Observation of apoptotic cells was carried out with a fluorescence microscope (Olympus, Tokyo, Japan) at a 350 nm excitation wavelength and 460 nm emission wavelength.

### Annexin V-FITC/PI staining to determine apoptosis

Apoptosis of MC3T3-E1 cells was determined using an Annexin V-FITC/PI Kit (Beyotime, Shanghai, China). In brief, cells were seeded into 6-well plates at 2.0 × 10^4^ cells/well. After treatment with 0, 0.6, 1.2, 1.8 and 2.4 mM FAC for 24 h, the cells were collected, incubated with Annexin V-FITC and PI following the manufacturer’s guidelines [24], and finally analyzed on a FACSCalibur flow cytometer (Becton Dickinson Immunocytometry Systems, San Jose, CA, USA).

### Western blotting

The total protein in cells was extracted and the concentration was assessed. Western blotting was conducted as described previously [25]. Antibodies against caspase 3, cleaved caspase-3, XIAP, c-IAP2, survivin, AKT, p-AKT, ERK, p-ERK, Stat3, p-Stat3, Smad4, and GAPDH were all obtained from Cell Signaling Technology Co., Ltd. (Danvers, MA, USA).

### miRNA profiling

The total RNA was isolated from MC3T3-E1 cells treated with or without 1.8 mM FAC for 24 h. miRNA next-generation sequencing analysis was carried out by BGI Genomics Co., Ltd (Shenzhen, China). Different expression of miRNAs between MC3T3-E1 cells treated without FAC (control group) and with FAC (FAC group) was identified through the log2Ratio (FAC/Control), which was calculated as the log2 value of the fold change, with threshold sets for up- and downregulated genes of a log2Ratio (FAC/Control) ≥ 1.0 or ≤ 1.0 with a *P* value < 0.05.

### qRT-PCR

The total RNA was isolated using TRIzol reagent (Keygen, Jiangsu, China). cDNA was synthesized from mRNA with the cDNA Synthesis SuperMix (Bimake, Houston, TX, USA) and from miRNA with a Reverse Transcription Kit (Ribo, Guangzhou, China). qPCR was performed to detect the expression levels of miR-3074-5p and Smad4 using the Bulge-Loop miRNA qRT-PCR Starter Kit (Ribo, Guangzhou, China) and 2 × SYBR Green qPCR Master Mix (Bimake, Houston, TX, USA) [26], respectively. The specific primers used are shown in Table 2. U6 and GAPDH were used as internal controls of miRNA and mRNA, respectively.

**Table 2:** Primer sequences for qRT-PCR

Gene	Sequences
miR-3074-5p	Forward: 5′-GCGGTTCCTGCTGAACTGA-3′
	Reverse: 5′-AGTGCAGGGTCCGAGGTATT-3′
Smad4	Forward: 5′-AGTTCACAATGAGCTTGCATTC-3′
	Reverse: 5′-TTCAAAGTAAGCAATGGAGCAC-3′
U6	Forward: 5′-TGCAGAGGATCTAATT-3′
	Reverse: 5′-GAAAGACCAGTCCAAGTCC-3′
GAPDH	Forward: 5′-TGTGTCCGTCGATCTGA-3′
	Reverse: 5′-TTGCTGTTTGTTGAAGTCGCAGGAG-3′

### Bioinformatics analysis

To understand the function of differentially expressed miRNAs in MC3T3 cells treated with or without FAC, KEGG pathway analysis was conducted to perform pathway enrichment analysis. In this study, enrichment factors of KEGG pathways in the top 20 were chosen and shown. Two bioinformatics software programs, TargetScan and miRanda, were used for target gene prediction. The intersection of their prediction results was taken as the final result.

### Dual-luciferase report gene assay

A wide-type 3ʹ-UTR fragment of *Smad4* mRNA (5ʹ…CUGUCAUGAGUGGAGCAGGAAG…3ʹ) containing a putative miR-3074-5p-binding site (position 2954–2960) was cloned into the psiCHECK_2_ vector, named WT-*Smad4*-3ʹ-UTR luciferase vector. The mutation of the 3ʹ-UTR fragment of *Smad4* mRNA (5ʹ…CUGUCAUGAGUGGAGCUCCAAG…3ʹ) was constructed using the QuickChange mutagenesis kit (Agilent Technologies, California, USA) and cloned into the psiCHECK_2_ vector to construct the MUT–*Smad4*-3ʹ-UTR luciferase vector [22]. HEK293T cells were transfected with psiCHECK2 reporter plasmids containing either the wild-type or mutant type of the 3ʹUTR of Smad4 in the presence of miR-3074-5p mimic and NC using Lipofectamine 2000, and cultured for 48 h before being harvested for experiments and analysis. The relative luciferase activity was implemented using the Dual-Luciferase Reporter Assay Kit (Yeasen, Shanghai, China).

### Statistical analysis

Quantitative data were expressed as the mean ± SD. SPSS 17.0 software (SPSS, Chicago, IL, USA) and GraphPad Prism 7.0 (GraphPad, San Diego, CA, USA) were used for statistical analysis. Independent samples *t* tests (unpaired *t*-tests) were performed for comparisons between two groups. One-way ANOVA was performed for comparisons among groups. *P* < 0.05 was considered to indicate statistical significance.

## Results

### Iron overload decreased the viability and induced apoptosis of MC3T3-E1 cells

**Fig. 1:**
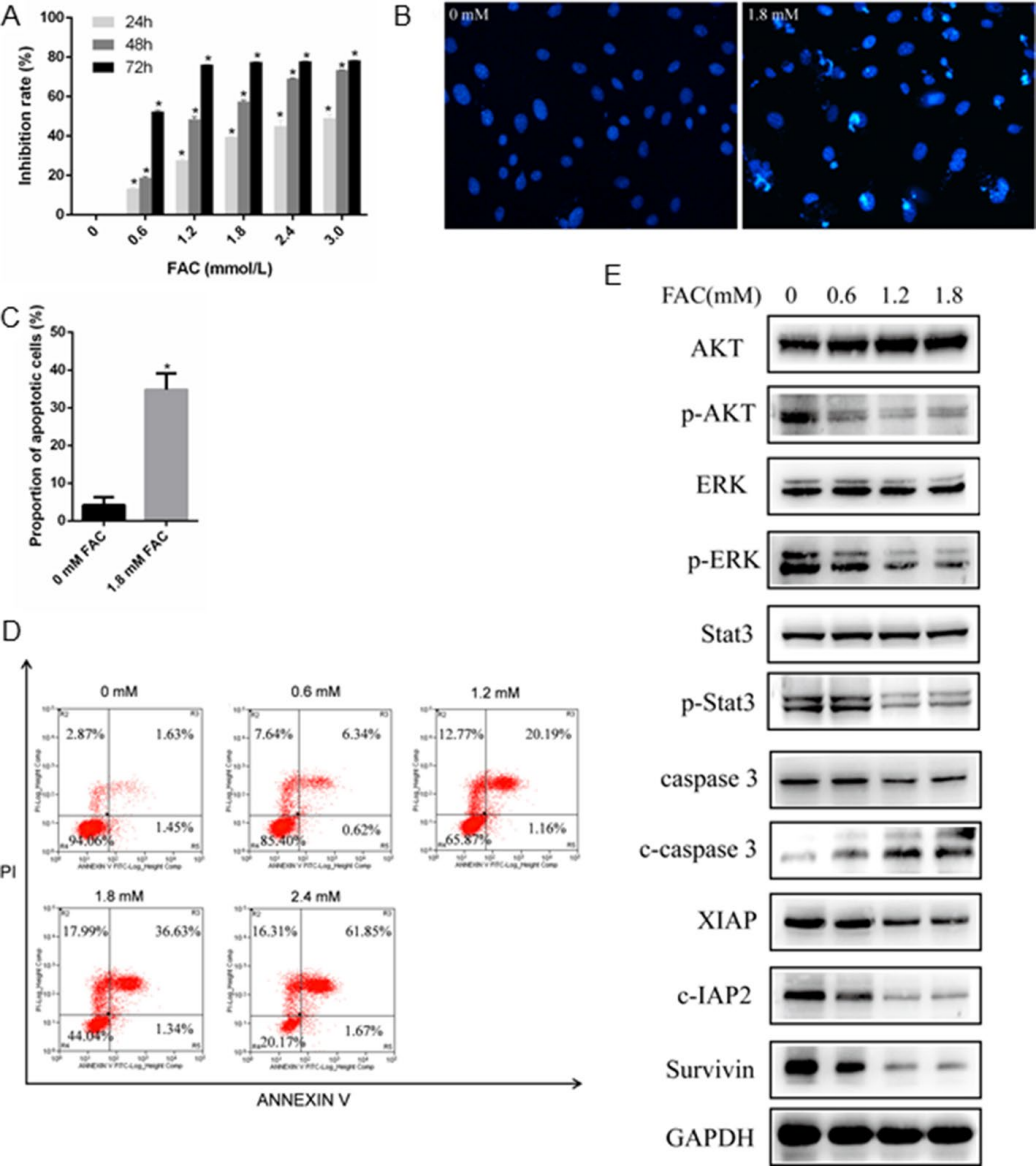
Iron overload decreased the viability and increased the apoptosis rate of MC3T3-E1 cells. **A** The viability of MC3T3-E1 cells was evaluated by CCK-8 assay after exposure to FAC (0–3.0 mM) for 24, 48 and 72 h. *P < 0.05 vs. the control. **B** MC3T3-E1 cells were treated with 0 and 1.8 mM FAC for 24 h. The nuclei of apoptotic cells treated with 1.8 mM FAC were densely stained with Hoechst 33,258, and were condensed and bright. Scale bars: 100 μm. **C** Quantitative analysis of apoptosis based on Hoechst 33,258 staining. *P < 0.05 vs. the control. **D** Cells were exposed to FAC (0–2.4 mM) for 24 h. Apoptotic MC3T3-E1 cells stained with Annexin V/PI were determined by flow cytometric analysis. **E** Apoptosis-related protein expression levels of cells were evaluated by western blot analysis after exposure to 0–1.8 mM FAC for 24 h

After exposing MC3T3-E1 cells to FAC (0, 0.6, 1.2, 1.8, 2.4 and 3.0 mM) separately for 24, 48 and 72 h, the CCK-8 assay revealed that FAC at each concentration inhibited the proliferation of MC3T3-E1 cells in a concentration- and time-dependent manner, compared with the untreated control group (*P* < 0.05) (Fig. 1a). To observe the morphological changes of FAC-treated MC3T3-E1 cells, cells treated with 0 and 1.8 mM FAC separately for 24 h were stained with a Hoechst 33,258 Staining Kit. The results of Hoechst staining showed that the nuclei of apoptotic MC3T3-E1 cells treated with 1.8 mM FAC were densely stained, which was the typical morphological change of apoptotic cells (Fig. 1b, c). To assess whether iron overload mediated cytotoxicity in MC3T3-E1 cells was related to apoptosis, Annexin V-FITC/PI staining was used to detect the apoptosis rate of MC3T3-E1 cells. Flow cytometry analysis showed that the late apoptosis rate increased from 1.63 to 61.85% as FAC concentrations increased from 0 mM to 2.4 mM, suggesting a dose-dependent effect of FAC on apoptosis in MC3T3-E1 cells (Fig. 1d). To confirm that iron overload did induce apoptosis of MC3T3-E1 cells at the protein level, the expression of apoptosis-related proteins in MC3T3-E1 cells was evaluated by western blot analysis. In this study, dose-dependent downregulation of XIAP, c-IAP2, survivin, p-AKT, p-ERK and p-Stat3, as well as upregulation of cleaved-caspase 3, was observed in MC3T3-E1 cells (0.6–1.8 mM FAC) for 24 h (Fig. 1e). All of the results demonstrate that iron overload promotes apoptosis of MC3T3-E1 cells.

### Iron overload induced miR-3074-5p expression in MC3T3-E1 cells

**Fig. 2:**
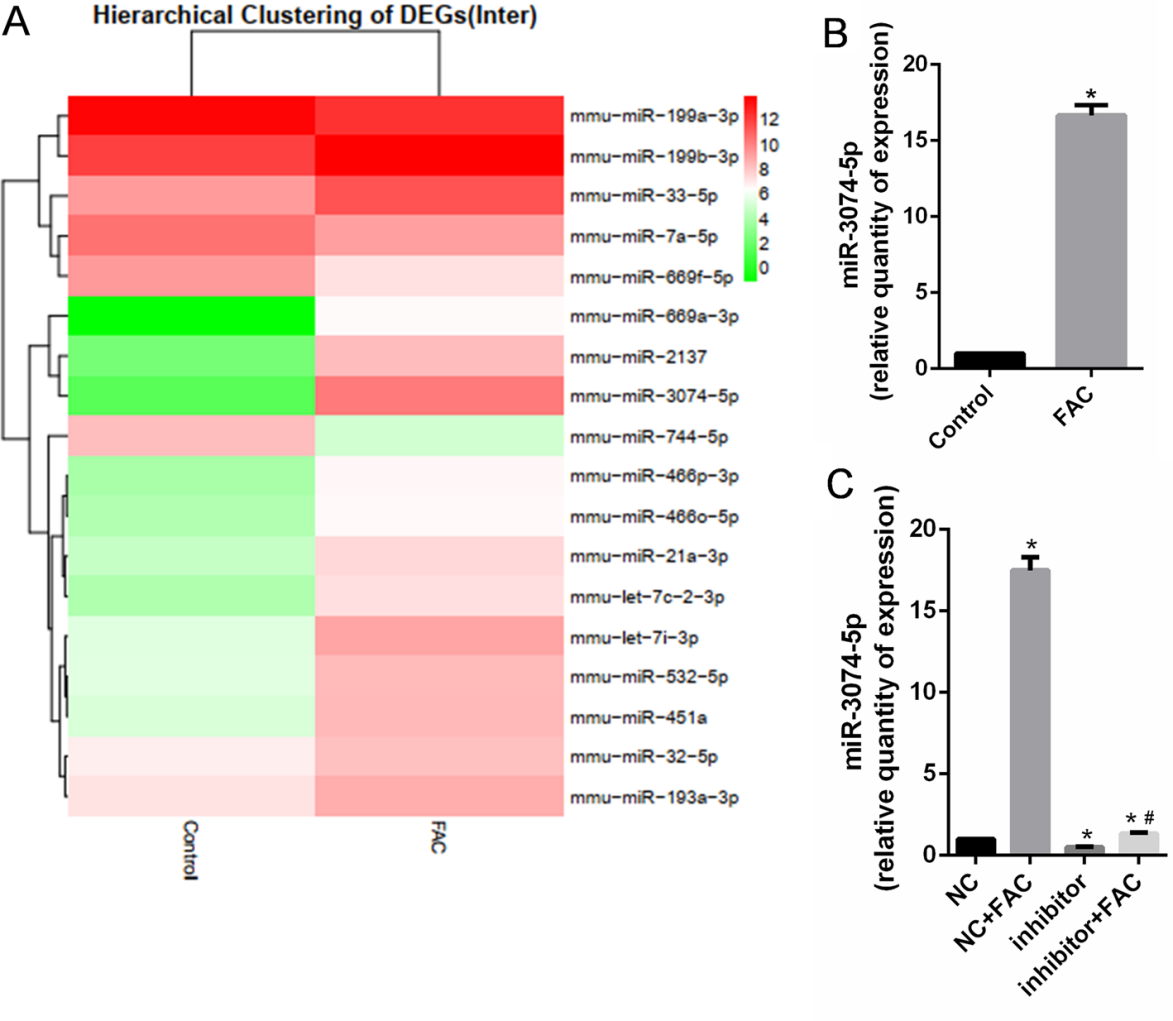
miR-3074-5p expression was increased in MC3T3-E1 cells under iron overload conditions. **A** Heatmap of 18 miRNAs displaying significant upregulation (red pixels) or downregulation (green pixels) in MC3T3-E1 cells treated with 1.8 mM FAC, compared with the control. **B**, **C** miR-3074-5p expression was validated by qRT-PCR. Inhibitor indicated miR-3074-5p inhibitor. NC indicates the negative control. *P < 0.05 vs. the NC group. ^#^*P* < 0.05 vs. the NC + FAC group

Differentially expressed miRNAs between MC3T3-E1 cells treated with and without 1.8 mM FAC for 24 h were identified via miRNA next-generation sequencing analysis (Fig. 2a). The raw data from the analysis have been deposited into the NCBI SRA database under the accession code PRJNA506880. We focused on miR-3074-5p, which displayed significant upregulation in iron-overloaded MC3T3-E1 cells, compared with the control group (*P* < 0.05). In order to validate this result, we examined the expression profile of miR-3074-5p in MC3T3-E1 cells treated with and without 1.8 mM FAC for 24 h. The qRT-PCR results showed that miR-3074-5p expression significantly increased in MC3T3-E1 cells exposed to FAC, compared to the untreated control group (*P* < 0.05) (Fig. 2b). We also found that cells transfected with miR-3074-5p inhibitor could rescue FAC-induced miR-3074-5p upregulation in MC3T3-E1 cells (Fig. 2c). The above results suggest a potential function of miR-3074-5p in MC3T3-E1 cells under iron overload.

### miR-3074-5p mediated iron overload-induced apoptosis of MC3T3-E1 cells

**Fig. 3:**
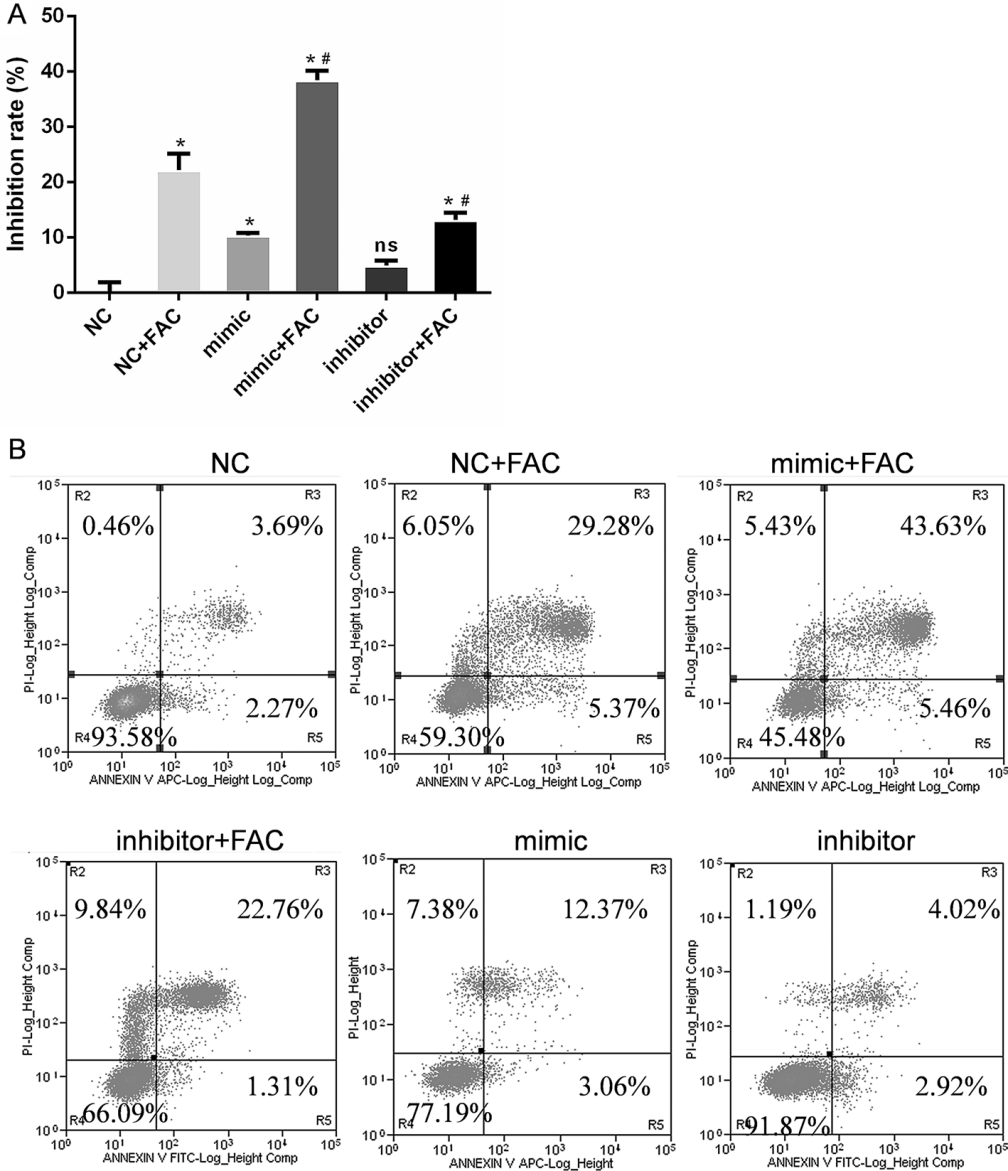
miR-3074-5p mediated the apoptosis of MC3T3-E1 cells under iron overload conditions. **A** Gain- and loss-of-function experiments were performed by transfecting cells with a miR-3074-5p mimic and miR-3074-5p inhibitor, respectively. The CCK-8 assay was used to investigate the effect of miR-3074-5p on cell viability. *P < 0.05 vs. the NC. ^#^*P* < 0.05 vs. the NC + FAC group. ns *P* > 0.05 vs. the NC group. FAC concentration: 1.8 mM. **B** Annexin V-FITC/PI staining was performed to confirm the function of miR-3074-5p in cell apoptosis. **C** Quantitative analysis of late apoptosis rate based on Annexin V-FITC/PI staining. *P < 0.05 vs. the NC. ^#^*P* < 0.05 vs. the NC + FAC group. ns *P* > 0.05 vs. the NC group

To explore the biological impact of miR-3074-5p on the apoptosis of MC3T3-E1 cells under iron overload conditions, gain- and loss-of-function experiments were performed by transfecting cells with a miR-3074-5p mimic or inhibitor, respectively. The CCK-8 assay was employed to define the effects of miR-3074-5p on cell viability and Annexin V-FITC/PI staining was performed to confirm the function of miR-3074-5p in cell apoptosis. The results revealed a significant increase in the inhibition rate of miR-3074-5p mimic-treated MC3T3-E1 cells under iron overload conditions (mimic + FAC group) compared with the negative control (NC)-treated MC3T3-E1 cells in the iron overload group (NC + FAC group) and the NC only group (*P* < 0.05). There was a significant decrease in the inhibition rate of miR-3074-5p inhibitor-treated MC3T3-E1 cells under iron overload (inhibitor + FAC group) compared with the negative control (NC)-treated MC3T3-E1 cells under iron overload (NC + FAC group) (*P* < 0.05) (Fig. 3a). Consistently, the apoptosis rate was increased in MC3T3-E1 cells transfected with the miR-3074-5p mimic under iron overload (mimic + FAC group) compared with the negative control (NC)-treated MC3T3-E1 cells in the iron overload group (NC + FAC group) and the NC only group (*P* < 0.05). Conversely, the apoptosis rate was decreased in cells that were transfected with the miR-3074-5p inhibitor under iron overload (inhibitor + FAC group), compared with the negative control (NC)-treated MC3T3-E1 cells under iron overload (NC + FAC group) (*P* < 0.05) (Fig. 3b). These results clearly indicate that aberrant expression of miR-3074-5p contributes to apoptosis of iron-overloaded MC3T3-E1 cells.

### Results of bioinformatic analysis

KEGG pathway analysis showed the target mRNAs of the differentially expressed miRNAs in MC3T3 cells treated with or without FAC. The top 20 enriched pathways with *P* < 0.05 were chosen and are shown (Additional file 1: Fig. S1a). Notably, these target genes of the differentially expressed miRNAs were enriched in two cellular survival- and death-related pathways, the FoxO signaling pathway and the mTOR signaling pathway (Additional file 1: Fig. S1a). Target mRNAs of miR-3074-5p were predicted with the miRanda and TargetScan databases. Eight hundred and sixty-four targets were identified by TargetScan and 450 targets were identified by miRanda; 314 targets overlapped between the two database. Among the predicted target genes, Smad4 was related to the FoxO signaling pathway and Jmjd8 was related to the mTOR signaling pathway (Additional file 1: Fig. S1b).

### miR-3074-5p targeted Smad4 in MC3T3-E1 cells

**Fig. 4:**
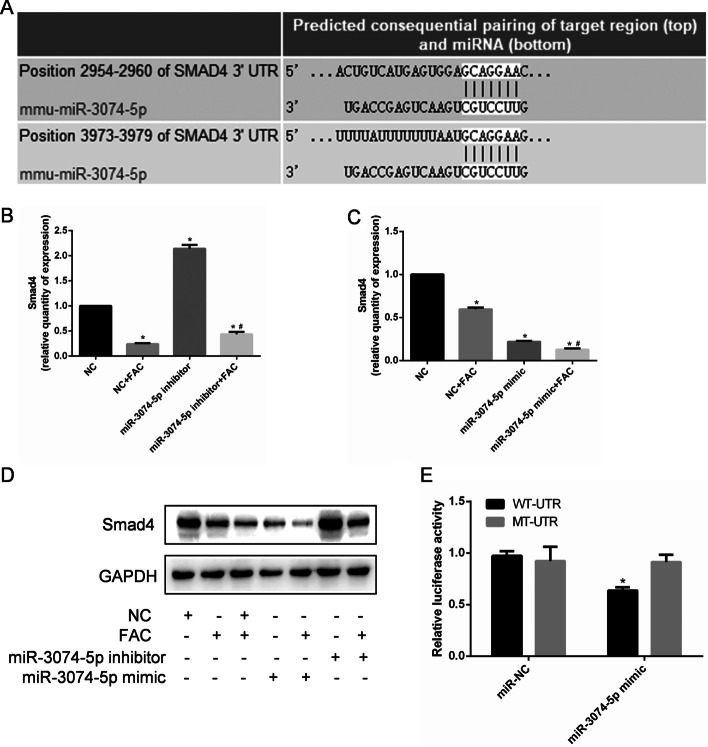
*Smad4* mRNA and protein were decreased in MC3T3-E1 cells under iron overload and were inversely correlated with miR-3074-5p expression. **A** Bioinformatics analysis showed that miR-3074-5p might bind to 3ʹUTR region of Smad4. **B** MC3T3-E1 cells were transfected with the miR-3074-5p inhibitor and NC separately and treated with or without 1.8 mM FAC. *Smad4* mRNA was quantified by qRT-PCR. **C** Cells were transfected with the miR-3074-5p mimic and NC separately and treated with or without 1.8 mM FAC. *Smad4* mRNA was quantified by qRT-PCR. *P < 0.05 vs. the NC group. ^#^*P* < 0.05 vs. the NC + FAC group. **D** MC3T3-E1 cells were transfected with miR-3074-5p mimic, miR-3074-5p inhibitor and negative control separately and treated with or without 1.8 mM FAC. Smad4 protein was detected by qRT-PCR. **E** The dual-luciferase report assay demonstrated that miR-3074-5p directly targets the 3ʹUTR of Smad4. The miR-3074-5p mimic repressed the activity of the wild-type (WT) Smad4 3ʹ-UTR (5ʹ…CUGUCAUGAGUGGAGCAGGAAG…3ʹ), but not that of the mutant type (MT) Smad4 3ʹ-UTR (5ʹ…CUGUCAUGAGUGGAGCUCCAAG…3ʹ). *P < 0.05 vs. the NC group

Further experiments are required to explore the molecular mechanisms linking miR-3074-5p with apoptosis of iron-overloaded MC3T3-E1 cells. Smad4 was identified as a potential target mRNA of miR-3074-5p. According to the results of bioinformatics analysis, miR-3074-5p might bind to 3ʹUTR of Smad4 (Fig. 4a). In this study, the expression of Smad4 in MC3T3-E1 cells under iron overload was lower than that in the untreated control group (*P* < 0.05) (Fig. 4b–d). The expression of *Smad4* mRNA and protein in MC3T3-E1 cells under iron overload was further reduced by overexpression of miR-3074-5p achieved by transfecting cells with a miR-3074-5p mimic, and the expression of *Smad4* mRNA and protein in MC3T3-E1 cells under iron overload was rescued by inhibition of miR-3074-5p by transfecting cells with a miR-3074-5p inhibitor (Fig. 4b–d), indicating that miR-3074-5p may mediate *Smad4* mRNA degradation by 3ʹUTR targeting. Moreover, miR-3074-5p regulates iron overload-promoted apoptosis in MC3T3-E1 cells by targeting Smad4, which was further confirmed by a dual-luciferase reporter assay (Fig. 4e).

### Smad4 attenuated miR-3074-5p mediated apoptosis in MC3T3-E1 cells induced by iron overload

**Fig. 5:**
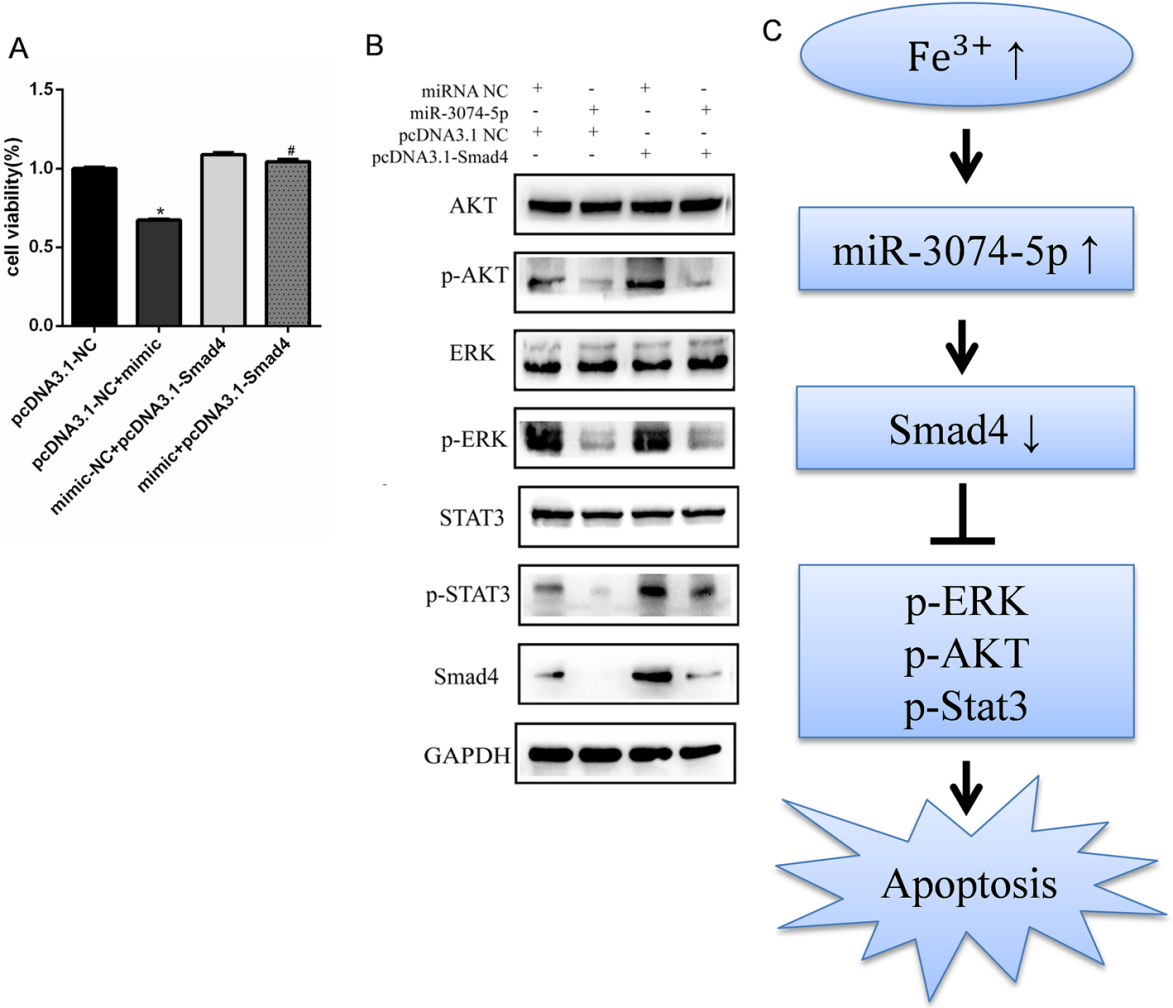
Restoration of Smad4 can rescue the effects of miR-3074-5p on MC3T3-E1 cells. **A** Gain- and loss-of-function experiments were performed by transfecting cells with pcDNA3.1-Smad4 and the miR-3074-5p mimic, respectively. The CCK-8 assay was used to evaluate the effect of Smad4 on cell viability. *P < 0.05 vs. the pcDNA3.1-NC. ^#^*P* < 0.05 vs. the pcDNA3.1-NC + mimic group. **B** Apoptosis-related protein expression levels of cells were evaluated by western blot analysis. **C** Working model of miR-3074-5p-mediated apoptosis in iron-overloaded MC3T3-E1 cells. miR-3074-5p expression was significantly increased in MC3T3-E1 cells under iron overload conditions. Overexpression of miR-3074-5p induced downregulation of Smad4, the knockdown of which inhibited ERK, AKT and Stat3 phosphorylation, resulting in apoptosis

Gain- and loss-of-function experiments were performed by transfecting cells with pcDNA3.1-Smad4 and miR-3074-5p mimic, respectively. The CCK-8 assay was employed to determine the effects of Smad4 on cell viability. The results revealed a significant increase in the inhibition rate of pcDNA3.1-NC + miR-3074-5p mimic-treated MC3T3-E1 cells (pcDNA3.1-NC + mimic group) compared with that of pcDNA3.1-NC-treated MC3T3-E1 cells (pcDNA3.1-NC group) (*P* < 0.05) (Fig. 5a). The inhibition rate of pcDNA3.1-Smad4 + miR-3074-5p mimic-treated MC3T3-E1 cells (pcDNA3.1-Smad4 + mimic group) was significantly lower than that of pcDNA3.1-NC + miR-3074-5p mimic-treated MC3T3-E1 cells (pcDNA3.1-NC + mimic group) (*P* < 0.05) (Fig. 5a). Consistently, the expression of pAKT, pERK, p-Stat3, and Smad4 was lower in MC3T3-E1 cells transfected with the pcDNA3.1-NC + miR-3074-5p mimic (pcDNA3.1-NC + mimic group), compared with the pcDNA3.1-NC-treated MC3T3-E1 cells (pcDNA3.1-NC group). Conversely, the expression of pAKT, pERK, p-Stat3, and Smad4 was higher in cells transfected with the pcDNA3.1-Smad4 + miR-3074-5p mimic (pcDNA3.1-Smad4 + mimic group) compared with the pcDNA3.1-NC + miR-3074-5p mimic-treated MC3T3-E1 cells (pcDNA3.1-NC + mimic group) (Fig. 5b). These results indicate that restoration of Smad4 can rescue the viability inhibition of MC3T3-E1 cells mediated by miR-3074-5p under iron overload.

## Discussion

Iron is a necessary auxiliary factor in many biological processes of the human body. Normally, iron metabolism is in a stable state in the human body. In osteoporosis patients, the internal environment of the bone marrow is in an iron overload state. Iron overload-induced osteoblast proliferation inhibition and osteoblast apoptosis are averse to bone remodeling in osteoporosis patients. In vivo and in vitro studies have shown that iron overload inhibits osteoblast proliferation and promotes osteoblast apoptosis. Iron overload induces osteoblast apoptosis by increasing ROS and activating the mitochondrial apoptosis pathway [10, 11]. However, the mechanism of activating the apoptosis pathway by targeting apoptosis-related genes via upstream microRNAs is still unclear. The purpose of this study was to analyze the miRNA expression profiles and evaluate the function of miR-3074-5p in the apoptosis of MC3T3-E1 cells under iron overload conditions and the potential mechanisms.

It has been reported that a high concentration of FAC could cause apoptosis of MC3T3-E1 cells [10, 25]. The results of this study showed that FAC at concentrations above 0.6 mM could induce apoptosis of MC3T3-E1 cells. Caspase 3 is known as one of the effectors of apoptosis. Endogenous, exogenous and endoplasmic reticulum pathways of apoptosis can ultimately activate caspase 3 through different signaling pathways and lead to apoptosis. In this study, the expression of caspase 3 decreased and that of cleaved-caspase 3 increased in FAC-treated MC3T3-E1 cells, suggesting that caspase 3 was activated and induced apoptosis. The IAP family is an inhibitor of apoptosis in cells, and includes XIAP, c-IAP1, c-IAP2, survivin, and NAIP. Among these proteins, c-IAP1 and 2 can inhibit the activation of caspase 9 by interfering with the Apaf-1/cytochrome C complex, thereby inhibiting the activation of caspase 3, while XIAP can directly inhibit the activation of caspase 3 [27]. Survivin can directly inhibit the activation of caspase 3 and caspase 9 [28]. AKT, ERK, and Stat3 play important roles in apoptosis and cell survival. They can inhibit apoptosis and promote cell survival by phosphorylation of themselves and inactivation of their downstream antiapoptotic targets. Apoptosis occurs when the phosphorylation of AKT, ERK, and Stat3 is blocked by stimulating factors [29–31]. In this study, the expression levels of XIAP, c-IAP2, and survivin in MC3T3-E1 cells under iron overload decreased, which promoted cell apoptosis.

In recent years, studies have shown that miR-1 regulates apoptosis of nitric oxide treated MC3T3-E1 cells by targeting HSP-70, and miR-335-5p inhibits the high glucose induced apoptosis of MC3T3-E1 cells, but the mechanism is not yet clear. The changes in the miRNA expression profile in the apoptosis of FAC-treated MC3T3-E1 cells and the mechanisms by which miRNAs regulate apoptosis of iron-overloaded MC3T3-E1 cells have not been studied. In this study, we confirmed the upregulation of miR-3074-5p in the apoptosis of MC3T3-E1 cells under iron overload conditions by next-generation sequencing and qRT-PCR. KEGG pathway analysis showed that miRNAs may regulate the apoptosis of iron-overloaded MC3T3-E1 cells by targeting apoptosis-related mRNAs associated with the FoxO and/or mTOR signaling pathways. Target mRNAs of miR-3074-5p were predicted by using bioinformatics analysis. Among the predicted target mRNAs, *Smad4* mRNA is related to the FoxO signaling pathway, and its downregulation can promote apoptosis. Previous studies have confirmed that the abrogation of Smad4 in chondrocytes and the blockade of Smad4 in cardiac myocytes result in increased apoptosis [32–34].

Smad4 is a key mediator in the signal transduction of transforming factor β (TGF-β)-Smad. Many previous studies have found that the BMP/Smad signaling pathway is involved in the regulation of osteogenic differentiation of osteoblasts. Inhibition of the expression of Smad1 and Smad4 can restrain the osteogenic differentiation of MC3T3-E1 cells [35, 36]. However, there are few studies on whether Smad4 is involved in the regulation of apoptosis in MC3T3-E1 cells. TGF-beta activates ERK in a Smad4-dependent manner and knockdown of Smad4 may inhibit ERK phosphorylation [37, 38]. ERK phosphorylation can promote the expression of antiapoptotic proteins such as Bcl-2 and inhibit the expression of proapoptotic proteins such as Bad. When ERK phosphorylation was blocked, these apoptosis inhibition effects were lost, and apoptosis was promoted. In addition, inhibition of ERK phosphorylation could cause activation of the FOXO transcription factor, leading to cell apoptosis [39]. It has been reported that activation of Smad4 can inhibit AKT phosphorylation and induce apoptosis of gastric cancer cells [40], but no relevant article has suggested that activation of Smad4 can block phosphorylation of Stat3 and lead to apoptosis. In this study, the expression of Smad4 in MC3T3-E1 cells under iron overload conditions was decreased, which could be reversed by inhibition of miR-3074-5p. Moreover, the expression of Smad4 in MC3T3-E1 cells under iron overload conditions was further reduced via overexpression of miR-3074-5p. These results suggested that miR-3074-5p may regulate the degradation of *Smad4* mRNA by targeting its 3ʹUTR, which was confirmed by the results of the dual-luciferase reporter assay.

In addition, it has been reported that iron overload can also inhibit the differentiation of MC3T3-E1 cells by inhibiting ALP activity and the expression of *colI* mRNA and protein [12], but its potential molecular mechanism and related signaling pathways still need to be further elucidated.

## Conclusions

In conclusion, this study showed that iron overload induced apoptosis in MC3T3-E1 cells, and miR-3074-5p is involved in viability inhibition of iron-overloaded MC3T3-E1 cells by directly targeting Smad4 (Fig. 5c). Further clinical analysis of miR-3074-5p-Smad4 in patients with osteoporosis should demonstrate the clinical relevance of this study, providing a new idea for solving the problem of bone loss in osteoporosis patients.

## Supplementary Information

**Additional file 1.** Results of bioinformatics analysis.

## Data Availability

All data generated or analyzed during this study are included in this manuscript.

## Abbreviations

AKT: Serine/threonine kinase
c-IAP2: Cellular inhibitor of apoptosis protein-2
ERK: Extracellular regulated MAP kinase
FAC: Ferric ammonium citrate
FoxO: Forkhead box O
miRNA: MicroRNA
mTOR: Mechanistic target of rapamycin kinase
MUT: Mutant
p-AKT: Phosphorylated AKT
p-ERK: Phosphorylated ERK
p-Stat3: Phosphorylated Stat3
qRT-PCR: Real-time quantitative reverse transcription PCR
Smad4: SMAD family member 4
Stat3: Signal transducer and activator of transcription 3
UTR: Untranslated region
WT: Wild type
XIAP: X-linked inhibitor of apoptosis
