# Supplementary material for: Apoptosis-promoting properties of miR-3074-5p in MC3T3-E1 cells under iron overload conditions
Source: Cell Mol Biol Lett. 2021 Aug 16;26:37. doi: 10.1186/s11658-021-00281-w (PMC8365891; doi:10.1186/s11658-021-00281-w)
Supplement: Supplementary file 1 — Additional file 1. Results of bioinformatics analysis. [file 11658_2021_281_MOESM1_ESM.docx]

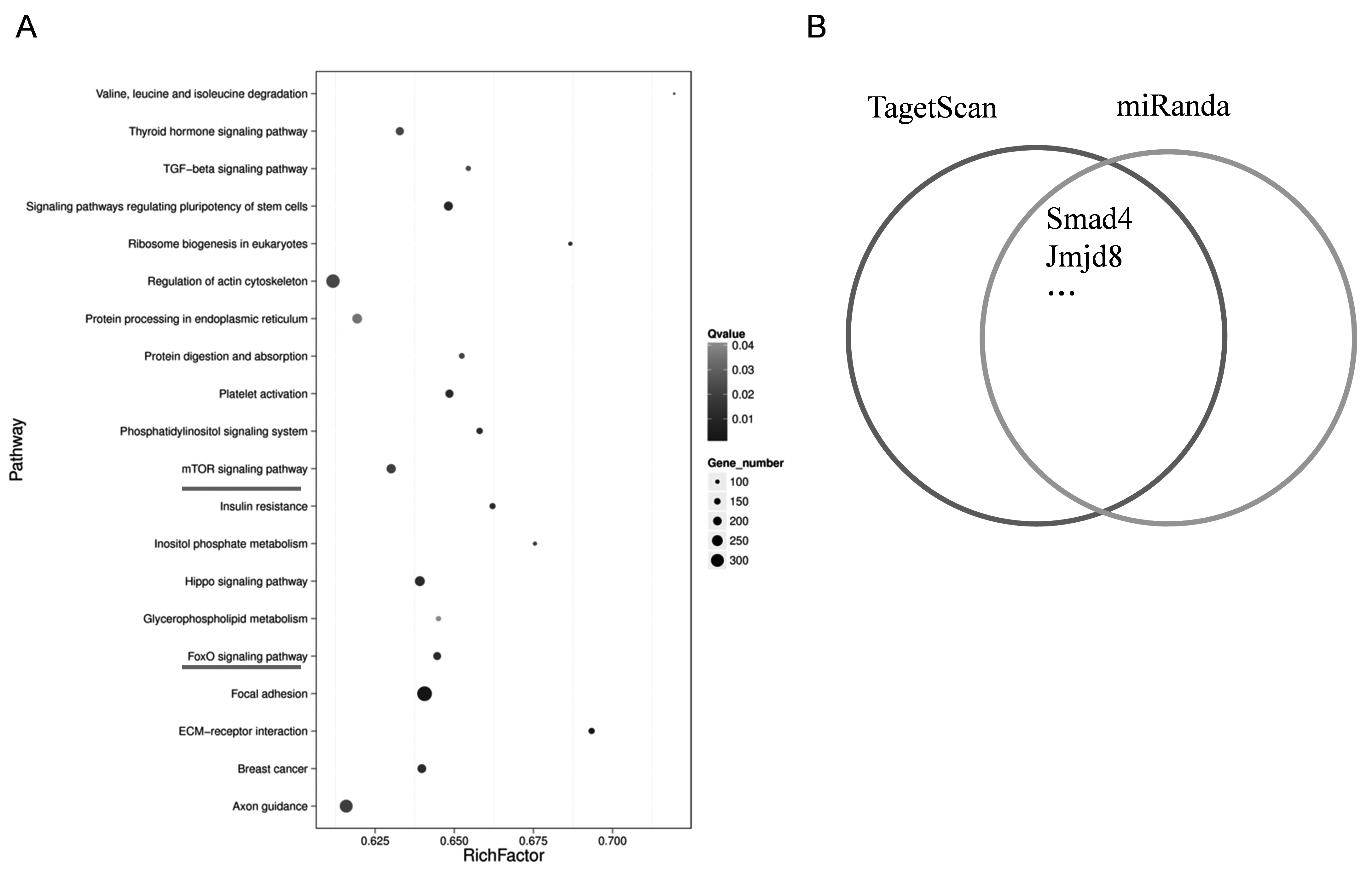


**Fig. S1** Results of bioinformatics analysis. **(**A) KEGG pathway analysis revealed that the target mRNAs of the differentially expressed miRNAs (DESs) in MC3T3 cells treated with or without 1.8 mM FAC. The top 20 enriched pathways with *P* < 0.05 were selected and are presented. The rich factor is the ratio of the DESs target gene numbers annotated in this pathway term to the total gene numbers annotated in this pathway term. The *Q*-value is the corrected *P* value, and ranges from 0-1. **(**B) Target mRNAs of miR-3074-5p were predicted with the miRanda and TargetScan databases.
